# Effects of intermittent fasting on brain health via the gut–brain axis

**DOI:** 10.3389/fnut.2025.1696733

**Published:** 2025-11-21

**Authors:** Ziqian Zhao, Wenting Geng, Yan Gao, Yitong Liu, Shanjing Nie, Qingqing Yin

**Affiliations:** 1 Department of Geriatric Neurology, Shandong Provincial Hospital Affiliated to Shandong First Medical University, Jinan, Shandong, China; 2 Institute of Brain Science and Brain-Inspired Research, Shandong First Medical University and Shandong Academy of Medical Sciences, Jinan, Shandong, China; 3 Shandong Academy of Occupational Health and Occupational Medicine, Shandong First Medical University and Shandong Academy of Medical Sciences, Jinan, Shandong, China

**Keywords:** intermittent fasting, brain health, gut-brain axis, microbial metabolites, neurodegenerative disorders, mental illness

## Abstract

Intermittent fasting (IF), an emerging dietary strategy alternating fasting and feeding cycles, exerts multi-modal brain protection through the regulation of the gut–brain axis. With neurological and mental disorders ranking among the top global disease burdens, IF opens new frontiers in nutritional neuroscience by modulating gut microbiota composition and metabolic pathways, offering a non-pharmacological intervention strategy. Preclinical studies reveal that IF enriches probiotics, reduces neuroinflammation, and restores intestinal barrier integrity, thereby mitigating “leaky gut”-induced cognitive decline. Similarly, the ketogenic effect of IF can improve mitochondrial efficiency, while its anti-inflammatory effect alleviates the pathological changes of multiple sclerosis by suppressing autoreactive T cells. Clinical evidence reveals that IF significantly correlates with decreased *β*-amyloid burden in Alzheimer’s disease (AD) transgenic models and enhanced motor performance in Parkinson’s disease (PD) patients, suggesting its multimodal neuroprotective effects. Mental health benefits are equally striking: IF rebalances the Firmicutes-to-Bacteroidetes ratio, which has been linked to anxiety and depression remission. The gut–brain axis (GBA) emerged as a pivotal mediator, with short-chain fatty acids (SCFAs) and tryptophan derivatives fostering serotonin synthesis and oxidative stress reduction. This review synthesizes preclinical and clinical evidence demonstrating how intermittent fasting modulates the gut–microbiota–metabolite–brain axis to promote neuroprotection and mental health benefits, while identifying personalized protocol optimization as a critical avenue for future research.

## 1 Introduction

Since the brain is the mediator of all our experiences and the agent of all our behaviors, brain health is critical for social wellbeing, productivity, creativity, and physical and mental health (1). However, there is no universally accepted definition of brain health. Nonetheless, according to the definitions provided by different researchers and organizations (1–5), brain health primarily consists of neurological health and mental health (Table 1). Neurological disorders are one of the leading causes of death globally, with 3.4 billion people affected by neurological health loss in 2021 and 11.1 million deaths attributed to neurological disorders (6). According to the 2019 Global Burden of Diseases, Injuries, and Risk Factors Study, mental illnesses continued to rank in the top 10 global sources of burden, with anxiety and depressive disorders being the most prevalent (7). Given that neurological and mental health disorders impose a growing global burden, there is a critical need for brain health prevention measures.

**Table 1:** Definitions of brain health.

Organizations or authors	Definition
World Health Organization	“The prevention of neurological disorders rests upon the promotion and development of optimal brain health across the life course. Good brain health is a state in which every individual can learn, realize their potential, and optimize their cognitive, psychological, neurophysiological, and behavioral responses while adapting to changing environments” (2), and “The state of brain functioning across cognitive, sensory, social–emotional, behavioral, and motor domains, allowing a person to realize their full potential over the life course, irrespective of the presence or absence of disorders.” The WHO recognizes that brain health encompasses neural development, plasticity, functioning, and recovery across the lifetime of an individual (3).
American Heart Association (AHA)	Optimal brain health is “an optimal capacity to function adaptively in the environment. This could be assessed in terms of competencies across the domains of thinking, moving, and feeling, encompassing, for example, the abilities to pay attention, perceive, and recognize sensory input; to learn and remember; to communicate; to problem solve and make decisions; to have mobility; and to regulate emotional status” (5).
Chen et al.	“A lifelong dynamic state of cognitive, emotional, and motor domains underpinned by physiological processes; it is multi-dimensional and can be objectively measured and subjectively experienced; brain health is influenced by eco-biopsychosocial determinants, resulting in a continuum of quality of life and wellness” (4).
Hachinski et al.	“A state of complete physical, mental, and social wellbeing through a full, balanced, continuous development and exercise of the brain” (1).

In recent years, dietary interventions have gained increasing attention as promising non-pharmacological approaches to support brain health. Various nutritional strategies, including caloric restriction (8), ketogenic diets (9), and specific nutrient supplementation (10), have demonstrated the potential for modulating neuroinflammation, enhancing synaptic plasticity, and promoting neuroprotection. Among these strategies, intermittent fasting (IF)—a dietary regimen alternating periods of fasting and feeding—is emerging as a novel non-pharmacological intervention for improving brain health (11). The GBA is a two-way communication network connecting the gut microbiota, metabolic pathways, and central nervous system function. According to preclinical research, IF improves the diversity of gut microbes, raises healthy metabolites such as short-chain fatty acids, and lowers systemic inflammation—all of which are important processes that affect neuroinflammation, synaptic plasticity, and cognitive resilience (12, 13). Activity-dependent brain-derived neurotrophic factor (BDNF) has emerged as a key regulator of cognitive performance and brain health. IF reduces oxidative stress and neuronal apoptosis by activating autophagy and upregulating neurotrophic factors (such as BDNF) (11, 14, 15). Furthermore, IF-induced ketogenesis regulates mitochondrial efficiency and energy metabolism in brain cells, which may postpone neurodegeneration (16). IF reduces neuroinflammation associated with “leaky gut,” which is a contributing factor to mental disorders and cognitive decline, by restoring the balance of the gut microbiota and strengthening the integrity of the intestinal barrier (17, 18). Additionally, the provision of neuroprotection effects and weight loss mediated by IF approaches may have a positive effect on mental health (19, 20). Its promise as a scalable intervention for optimizing brain health is highlighted by the association between IF, gut microbiota, and brain function. This review aims to investigate the mechanisms linking IF to brain health, with an emphasis on the function of the GBA.

## 2 Brain health and gut microbiota

Numerous chemical signals from the environment are sensed, altered, and adjusted by the gut microbiota, which functions as a filter and biological rheostat. These signals then travel throughout the body and may have a direct impact on human health. Through immunological, endocrine, and neurological signaling pathways, the gut microbiota interacts with the central nervous system (CNS). By activating sympathetic and parasympathetic neurons in the gut (21), educating the immune system (22), and regulating the synthesis of various neurotransmitters and gut toxins (23, 24), the gut microbiota communicates with the brain via the aforementioned networks. Additionally, several gut microbial metabolites of interest, such as known neuromodulators (25), pro-inflammatory and anti-inflammatory mediators (26), and molecules that energize host cellular metabolism (27), can be implicated in brain function, such as blood–brain barrier (BBB) integrity and the regulation of neurodevelopment and neuroinflammation. To provide deeper mechanistic insights, we currently present a stepwise explanation of key pathways through which microbial metabolites influence brain health. Specifically, butyrate—a major short-chain fatty acid produced by gut microbiota—crosses the BBB via monocarboxylate transporters and exerts neuroprotective effects through histone deacetylase (HDAC) inhibition (28). This inhibition leads to the increased acetylation of histones surrounding the BDNF promoter, thereby enhancing BDNF expression and promoting synaptic plasticity (29). Additionally, butyrate modulates microglial activation and reduces neuroinflammation through G-protein-coupled receptor signaling pathways, representing a crucial mechanism linking gut microbiota to brain health (30). Through hormones and neuroactive substances, changes in the intestinal microenvironment mediated by the gut microbiota are indirectly transferred from the gut’s immunological and epithelial cells to enteric nervous system cells, where they are converted into neural impulses that impact the CNS (31). The so-called “GBA,” or gut–brain signaling, has been linked to mental and neurological disorders. While this review has primarily focused on GBA signaling, it is important to emphasize that the GBA operates in a bidirectional manner. Central processes significantly influence gut physiology and the microbiota composition through the neuroendocrine and autonomic pathways. For instance, psychological stress activates the hypothalamic–pituitary–adrenal axis, leading to increased cortisol release that can alter gut permeability, modify intestinal motility, and change the microbial composition (32). Similarly, emotional states and neurological conditions can affect gut function through sympathetic nervous system activation, creating a feedback loop that may exacerbate both gastrointestinal and neurological symptoms (6, 33, 34). This bidirectional communication underscores the complexity of the axis and highlights how brain states can profoundly influence the gut microenvironment, which, in turn, feeds back to affect brain health. Conditions such as depression, autism spectrum disorder, Parkinson’s disease, and Alzheimer’s disease have been linked to changes in gut microbiota taxonomy and microbial metabolites (35). Thus, by influencing the gut–brain axis, it may be possible to prevent and treat neurological and mental disorders by modifying specific types of neuroactive metabolites and neuronal transmission. IF may promote gut–brain information exchange by modifying the composition of the gut microbiota and microbial metabolism, which, in turn, exerts neuroprotective effects.

## 3 IF improves brain health through the GBA

IF is defined as a dietary pattern that restricts the timing of eating rather than the amount or composition of food, while ensuring the absence of malnutrition. IF regimens are diverse, and the majority of IF protocols described in the scientific literature fall into five categories: alternate day fasting (ADF), alternate day modified fasting, time-restricted feeding (TRF), fasting mimicking diets (FMD), and periodic fasting. While these IF protocols share common metabolic benefits, emerging evidence suggests that they may engage distinct mechanisms through the gut–brain axis. TRF, typically involving 8–12 h feeding windows, primarily affects the circadian rhythms of the gut microbiota and enhances the microbial diversity without significant caloric restriction (36). ADF, which involves 24-h fasting periods alternating with *ad libitum* feeding days, induces more pronounced metabolic switching and ketone production, potentially exerting stronger effects on mitochondrial biogenesis (37). FMD is an emerging dietary pattern characterized by caloric restriction and limited intake of protein from animal sources, and it may preferentially modulate immune function and inflammatory pathways through profound gut microbiota restructuring (38, 39). The divergent outcomes observed in IF studies may be attributable to protocol-specific effects, underscoring the critical need to account for methodological variations when interpreting their impact on brain health. The duration of IF interventions also appears to play a crucial role in determining outcomes. Short-term IF (8 weeks) primarily improves gut barrier function and reduces systemic inflammation (40), whereas longer interventions (12 weeks) exert more substantial effects on neurotrophic factors and cognitive performance (41). The efficacy of IF is not uniform but is significantly modulated by factors such as disease models and subject characteristics. For instance, ADF demonstrates particular efficacy in metabolic disorders, while TRF appears more beneficial for neurological conditions associated with circadian rhythm disruptions (37, 42). Future studies should systematically compare these protocols head-to-head to establish their relative efficacies for specific brain health applications.

**Figure 1:**
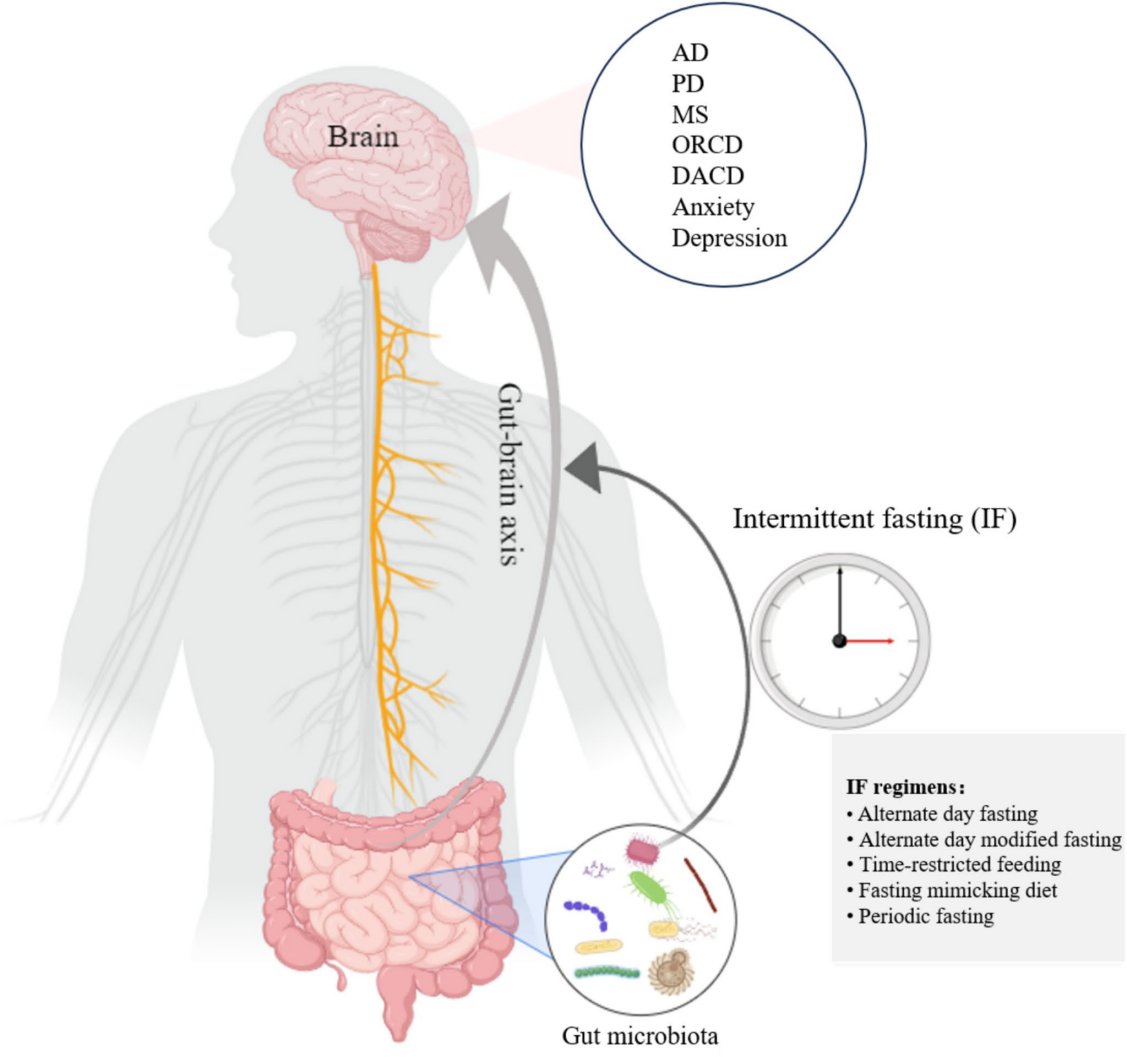
IF establishes gut–brain information exchange by influencing the gut microbiota, which in turn exerts a brain-protective effect. This figure was created on Medpeer.cn.

After the body is challenged by fasting-induced energy deprivation for 12–36 h, a distinct metabolic transition, or “switch,” occurs in cells, shifting from glucose and carbohydrate utilization to fatty acids and ketones as primary fuel sources (20, 43). The metabolic pattern of the cell alternates between the fed and fasting states, and this alternation is central to IF’s mechanism (20). Recent research has significantly advanced our understanding of the metabolic effects of various IF protocols. For instance, TRF has been demonstrated to restore diurnal fluctuations in gut microbiota composition and enhance microbial diversity, even under isocaloric conditions (44). This restoration of microbial circadian rhythms promotes overall metabolic health. Furthermore, TRF induces a metabolic switch toward fatty acid oxidation, increasing ketone body production and improving mitochondrial function (45). These adaptations are particularly significant for brain health, as ketones serve as an alternative energy substrate for neurons and possess neuroprotective properties. The synchronization of feeding-fasting cycles with microbial circadian rhythms, therefore, represents a fundamental mechanism through which IF confers its benefits via the GBA. Evidence from animal studies supports the protective effects of IF on various brain-related diseases, including AD (46), PD (47), multiple sclerosis (MS) (48), and mental disorders (49, 50). The gut–brain axis is a key pathway that mediates the impact of IF on brain health. Dynamically oscillating microbiota are believed to adapt and respond to environmental changes during diurnal fluctuations (51). Notably, when nutritional intake remains unchanged, time-restricted feeding can restore these cyclic fluctuations, thereby enhancing gut microbiota diversity (44). Liu et al. found that IF altered microbial metabolites and enriched the composition of the gut microbiome, which improved cognitive functions, such as in spatial memory tasks (18). Given the growing relationship between gut microbiota and brain health and the possibility that IF regulates gut microbiota, it is helpful to investigate how IF affects brain health and how the gut–brain axis plays a role in this process. The primary aim of this opinion article is to analyze the recent advances in IF in enhancing brain health, with a particular emphasis on the gut–brain axis pathway (Figure 1).

### 3.1 Neurodegenerative disorders and IF

#### 3.1.1 Alzheimer’s disease

Alzheimer’s disease (AD) is a progressive neurodegenerative disease characterized by memory decline and cognitive impairment, and the number of AD patients is increasing as a result of population aging and worldwide population expansion (52). AD is a by-product of several risk factors, such as neurofibrillary tangles and excessive deposition of amyloid plaques (53), oxidative stress (54), neuroinflammation (55), and neurotransmitter imbalances (56). In a transgenic AD mouse model, new evidence indicates that IF improves cognitive functions and AD-like pathology by altering the composition of the gut microbiota, with a notable enrichment in probiotics such as Lactobacillus, decreased carbohydrate metabolism (such as glucose), and increased abundance of amino acids (such as sarcosine and dimethylglycine) (57). Amyloid precursor protein, the precursor of Aβ, has been demonstrated to decrease in the blood of 14 healthy subjects after 30 days of IF in patients with AD or mild cognitive impairment (58). Compared to age-matched controls with mild cognitive impairment (MCI) who did not practice intermittent fasting, IF improved cognitive functioning in older adults with MCI over a 3-year period (59). Further mechanistic investigations reveal that the neuroprotective effects of IF are mediated through multi-level pathways. As previously discussed, butyric acid—a short-chain fatty acid produced by gut bacteria during IF—can cross the BBB and inhibit HDAC activity, thereby upregulating BDNF expression. Elevated BDNF levels increase the proliferation of primary adult hippocampal neural stem cells and embryonic cortical neural stem cells in mice by activating TrkB receptors, while reducing A*β*-induced cell death (60). This ultimately promotes neuronal survival, synaptic plasticity, and reduces β-amyloid toxicity.

#### 3.1.2 Parkinson’s disease

Parkinson’s disease (PD), another common neurodegenerative disease in the elderly, is characterized by Lewy bodies of *α*-synuclein in the brain and dopamine neuron depletion or impairment in the substantia nigra regions, which leads to uncontrolled striatal neuron discharge and subsequent cognitive decline (61, 62). The PD community is widely aware that nutrition plays a significant role in the illness. In particular, by altering the gut microbiome, the IF regimen reduced neuroinflammation in PD model mice, resulting in improved motor skill retention and fewer dopaminergic cell losses in the substantia nigra (63).

#### 3.1.3 Multiple sclerosis

Although it is commonly classified as an autoimmune illness, multiple sclerosis (MS) is a disease of the central nervous system that is characterized by demyelination and neurodegeneration that are mediated by both T and B cells. Clinically, MS patients have deficiencies in executive functioning, long-term memory, processing speed, complicated attention, and information processing efficiency (64). Alternate day fasting for 4 weeks triggered microbial metabolic pathways and enhanced gut microbiota richness in an MS animal model, suggesting that IF may influence MS impairment in animal models through gut microbiota modification (65). This, in turn, resulted in decreased T-lymphocyte numbers, which are believed to be responsible for the pathophysiology of MS (66). Interestingly, transplantation of the gut microbiota from MS mice on an IF diet reduced MS pathogenesis in recipient MS mice without an IF diet (65). Recent clinical investigations further support these findings. A 2023 randomized controlled trial demonstrated that time-restricted feeding (16:8 protocol) for 8 weeks significantly reduced neuroinflammatory markers and improved fatigue, sleep quality, and overall health status in patients with relapsing–remitting MS (67). Additionally, dietary restriction regimens that utilize continuous or intermittent food restriction can induce anti-inflammatory, immunomodulatory, and neuroendocrine adaptations, exerting neuroprotective effects (68).

### 3.2 Metabolic neurological disorders and IF

A major hazard to public health is obesity-related cognitive dysfunction (ORCD), a worldwide epidemic that is frequently linked to cognitive deterioration in various groups (69, 70). IF is a promising strategy for alleviating obesity and its related metabolic health consequences (61, 71, 72). One important regulator of neural function is the microbiota–gut–brain axis. Through the gut–brain axis, IF reduces obesity-related cognitive impairment and results in clinically substantial weight loss (61, 73, 74). A recent animal study demonstrated that IF alleviated ORCD, especially during weight-loss and weight-regain periods, by promoting the generation of short-chain fatty acids and modulating the gut flora (12).

Type 2 diabetes is becoming more commonplace worldwide. Diabetes-associated cognitive dysfunction, which is a high-prevalence comorbidity in diabetics, shows up clinically as accelerated neurodegeneration, executive dysfunction with attentional deficits, and progressive episodic memory deterioration (75, 76). A 28-day IF regimen for diabetic mice has been shown in recent studies to alleviate behavioral impairment through a microbiota–metabolites–brain axis: IF restructures the gut microbiota, improves microbial metabolites linked to cognitive function, and increases the expression of genes involved in energy metabolism and mitochondrial biogenesis in the hippocampus (18).

### 3.3 Mental illness and IF

One of the Sustainable Development Goals of the UN is mental health, and mental illnesses mostly include depression, anxiety, and other conditions (77). According to the World Health Organization (87), the yearly global loss in productivity resulting from anxiety and depression disorders is US$1 trillion, and this figure is predicted to increase (78). Numerous mental illnesses, including anxiety and depression, have been linked to the makeup and abundance of the gut microbiota, particularly Firmicutes and Bacteroidetes (79, 80). Mice and patients with anxiety typically exhibited higher levels of Bacteroidetes and Fusobacteria but lower levels of Firmicutes at the phylum level. Preclinical models show that animals with anxiety- and depressive-like behaviors have gut microbiota disruptions and that bacterial probiotic treatment normalizes both behavioral and microbial changes (81, 82). At the phylum level, IF demonstrated the capacity to alter the Firmicutes-to-Bacteroidetes ratio, resulting in higher Firmicutes (83) and lower Bacteroidetes (84), thereby remodeling the gut microbiota. Overall, there is a strong hypothesis that IF regimens could improve mental health by influencing the gut–brain axis. Additionally, Fond G et al. observed that IF, or fasting for 12–16 h a day, improves microbiota and, consequently, mental health issues (85). New longitudinal cohort studies have strengthened this connection. A randomized controlled trial by Jamshed et al. found that intermittent fasting was associated with significant reductions in weight and body fat, as well as marked improvements in fatigue, physical strength, and depressive symptoms (86). Notably, interventions extending beyond 12 weeks yielded superior outcomes on the emotional assessment metric.

## 4 Conclusion and future directions

The GBA plays a significant role in brain health, highlighting the potential of IF as a non-pharmacological strategy for managing related disorders. Translating this potential into clinical practice paves the way for precision nutrition, where IF regimens can be personalized based on an individual’s metabolic profile, neurological status, and gut microbiota composition. Advanced technologies—including gut–brain organoids, artificial intelligence-driven analytics, and CRISPR-engineered probiotics—offer promising tools for predicting therapeutic responses and personalizing interventions. Ultimately, realizing this vision will require enhanced multidisciplinary collaboration among neuroscientists, microbiologists, and nutritionists to bridge fundamental research and clinical application.

Nevertheless, a critical appraisal of the current evidence reveals important limitations that temper this optimistic outlook. A primary concern is the field’s substantial reliance on preclinical models. While these studies provide invaluable mechanistic insights, they often fail to fully capture the complexity of human physiology and long-term outcomes. This gap underscores the imperative for rigorous, large-scale human trials to definitively establish the safety and efficacy of intermittent fasting across diverse populations. Moreover, IF is not a universally applicable intervention and carries potential risks for specific groups, such as adolescents, individuals with a history of eating disorders, pregnant women, and those with specific metabolic conditions. Adverse effects may include nutrient deficiencies, hormonal disruptions, and the exacerbation of disordered eating patterns.

Therefore, a balanced perspective is essential for the responsible advancement of IF research. While the mechanistic insights through the gut–brain axis are compelling, future studies must prioritize identifying biomarkers that predict individual responses and establishing clear safety guidelines for vulnerable populations. The ultimate challenge lies not only in validating the efficacy of IF but also in developing personalized approaches that maximize benefits while minimizing potential harms and in addressing the practical challenges of long-term adherence and sustainability in real-world settings. Thus, a clear-eyed acknowledgment of these limitations constitutes a critical prerequisite for the safe and effective translation of IF into clinical practice.
